# Metasynthesis of the Views about Treatment of Anorexia Nervosa in Adolescents: Perspectives of Adolescents, Parents, and Professionals

**DOI:** 10.1371/journal.pone.0169493

**Published:** 2017-01-05

**Authors:** Jordan Sibeoni, Massimiliano Orri, Marie Valentin, Marc-Antoine Podlipski, Stephanie Colin, Jerome Pradere, Anne Revah-Levy

**Affiliations:** 1 Service Universitaire de Psychiatrie de l’Adolescent, Argenteuil Hospital Centre, Argenteuil, France; 2 ECSTRA Team, UMR-1153, Inserm, Paris Diderot University, Sorbonne Paris Cite, Paris, France; 3 Centre Hospitalier du Rouvray, Fédération Hospitalo-Universitaire de Psychiatrie de l'Enfant et de l'Adolescent, CHU de Rouen - CH du Rouvray, Rouen, France; Hospital Universitari de Bellvitge, SPAIN

## Abstract

**Background:**

Anorexia nervosa in adolescents can be a difficult-to-treat disease. Because qualitative research is a well-established method for deepening our understanding of subjective experiences, such as eating disorders and their treatment, we sought to perform a systematic review of qualitative studies to synthesize the views of adolescents with this disease, their parents, and their healthcare providers about its treatment.

**Methods:**

We performed a thematic synthesis to develop the central themes that summarize all of the topics raised in the articles included in our review. The quality of the articles was assessed by the Critical Appraisal Skills Program.

**Results:**

We included 32 articles from seven different countries. Two central themes were inductively developed from the analysis: (1) the treatment targets (i.e., symptoms and patients in context), and (2) a therapeutic tool—a relationship, specifically the core concept of the therapeutic relationship.

**Conclusion:**

Our results underline the difficulty in establishing a therapeutic alliance, the barriers to it, especially the risk that professionals, adolescents, and parents will not converse about treatment; although such a dialogue appears to be an essential component in the construction of a therapeutic alliance.

## Introduction

Anorexia nervosa (AN) is a disease characterized by a distorted body image and restricted food intake that leads to severe weight loss. People with AN most often develop this disorder during adolescence [1]. During this period, the mortality rate is high and somatic and psychiatric complications frequent [2–4]. In fact, the treatment trajectory of adolescents with AN may have a chronic or relapsing course, with episodes of dropping-out of treatment [5]. On the one hand, adolescents with AN are described as resistant to the treatment due to the ego syntonic nature of the disease [6]; on the other hand, treatment programs that fail to be tolerable to patients result in poor adherence and treatment retention. One community-based study prospectively examined the long-term outcome of 51 adolescents with AN and found that, after 18 years, 12% percent of the sample still had an eating disorder and 39% of them a psychiatric disorder other than an eating disorder [7]. Somewhat better results came from a longitudinal prospective study of adolescents with severe AN who underwent inpatient treatment; Strober et al. [5] found that by 10 to 15 years after discharge, 76% of this clinical sample had achieved full recovery but noted that 29.5% relapsed following hospital discharge. Thus, these studies investigating long-term outcome report relatively good recovery rates but underline the issues of chronicity, relapse, and co-morbid disorders [5,7].

More recently, different therapeutic approaches have been recognized as effective treatments for adolescent AN, including enhanced cognitive behavior therapy [8] or family based-treatment (FBT) [9]. The latter has developed evidence that justifies its recommendation in clinical practice guidelines [10]. Yet, specific aspects of the treatment of adolescents with AN remain the topic of ongoing debates, including the superiority of FBT and the efficacy of various settings, such as day hospital or full-time inpatient hospitalization [11]. For example, the multicenter study by Herpertz-Dahlmann et al. [12] about treatment settings recently reported that day hospital treatment is not inferior to full-time inpatient admission for the full period in adolescents with AN.

Many qualitative studies have addressed various issues about treatment for AN in adolescence with the objective of improving the quality of care, especially by studying the process of treatment. In contrast to quantitative studies of efficacy, which aim to evaluate outcomes in terms of reductions in scores on measures of eating disorder pathology, qualitative research seeks to describe and deepen our understanding of complex issues around treatment from the perspective of participants—for example the perspectives of patients, family members or health professionals-. Qualitative studies about adolescent AN thus give us access to views about treatment from the points of view of all the stakeholders.

We chose to apply an approach based on metasynthesis in order to transform the initial findings of original qualitative studies into decontextualized results that are more abstract and generalizable [13–15]. Metasynthesis is a systematic review of the literature of qualitative studies on a subject [16]; its aim is to “achieve analytical abstraction at a higher level, by rigorously examining the overlapping elements in common among studies” [17]. It has a twofold objective: not only to summarize the existing qualitative publications on a given subject, but also to open new interpretive pathways by their comparison and joint analysis. Over the last five years, metasynthesis has been shown to be a useful tool in psychiatry and medical research [18].

To date, two metasyntheses on the treatment of AN have been published, both focusing on the experience of patients. Espindola and Blay [19] explored the treatment of AN from the views of patients—both adolescents and adults. They selected and analyzed 16 studies and described original concepts of recovery, especially self-acceptance and self-reconciliation. Bezance and Holliday [20], who selected and analyzed 11 articles, investigated the perspective of adolescents with AN. They underlined the importance of family involvement in care, of peer groups, and of the need for a comprehensive bio-psycho-social approach. Other qualitative studies—included in our metasynthesis—have also explored the views of parents or of health-care providers, but they have not been included in the previous metasyntheses. Crossing the perspectives of patients, families, and healthcare providers enables a better understanding of their shared representations of the disease and its treatment. In recent years we have conducted several qualitative studies exploring the intersecting viewpoints around the issues of obesity [21] and attempted suicide [22] in pediatric populations as well as on the topics of cancer [23] and AN [24] in adults.

In line with these previous studies, we performed a metasynthesis of the qualitative studies exploring the views about treatment of AN in adolescence from the perspectives of the adolescents, their families, and finally the professionals who treat them. Our objective was to describe, compare, and contrast these three perspectives to generate new insights about the issues around treatment of adolescent AN that might have new clinical implications that could improve the care process.

## Methods

### Study design

This metasynthesis relies on the model of meta-ethnography [15] and follows the procedures of the thematic synthesis described by Thomas & Harden [14].

It complied with the ENTREQ guidelines [18].

It consisted of six successive stages:

1. Defining the research question, the subjects, and the types of studies to be included
2. Identifying and selecting the studies
3. Assessing the quality of the studies selected
4. Analyzing the studies, identifying their themes, and translating these themes between the studies
5. Generating the themes of the analysis and structuring the synthesis
6. Writing the synthesis.

The thematic analysis contained two phases: one descriptive, which defined and compared the themes, and the other interpretive, which developed original ideas drawn from the review.

### Search strategy and selection criteria

We conducted a systematic search in five databases according to a search algorithm specific to each base: Medline, PsycINFO, CINAHL, EMBASE, and SSCI. The inclusion and exclusion criteria were debated at meetings of our research group, composed of specialists in qualitative research and in eating disorders in adolescents.

Inclusion criteria: Only studies using a qualitative methodology, published in English (as most studies are now published in English) from 1990 to 2015 (as qualitative health research has developed mainly over these past 25 years), concerning the treatment of AN (all forms of AN: pure restriction, binge and purge) in adolescence. Participants could be patients (younger than 18 years during their disease), their families, or the healthcare professionals caring for them.

The exclusion criteria resulted in the non-inclusion of studies using quantitative or mixed methodology or concerning nonspecific eating disorders, mixed eating disorders, or bulimia nervosa.

The study took place from March 2014 through September 2015. Preliminary research had identified several articles from which we selected key words. The research group used existing literature reviews to determine a list of key words, a mix of *free-text terms* and *thesaurus terms*, referring to AN, adolescence, and qualitative research, to collect studies indexed in the databases (Table 1). The focus of our metasynthesis was treatment, but we decided not to use key words referring to it because numerous qualitative studies discuss treatment or consider its implications without mentioning it explicitly in either the title or the abstract. We performed the literature search on March 5, 2014, with one update, on September 1, 2015. Complete search strategy for each database is available in the supplemental material (S1 Table).

**Table 1:** Result of search strategy for each database.

Database	Dates	Result
CINAHL Plus (EBSCO Publishing) [1981–]	01/01/1990 to 09/01/2015	512
Embase (Ovid) [1974–]	01/01/1990 to 09/01/2015	275
Medline (PubMed) [1948–]	01/01/1990 to 09/01/2015	63
PsycINFO (EBSCO Publishing) [1800–]	01/01/1990 to 09/01/2015	57
SSCI (Web of Science) [1956–]	01/01/1990 to 09/01/2015	806

After collecting the references and eliminating duplicates, two authors (JS and MO) subsequently read the titles and abstracts to assess their relevance to our target subject and methodology. The database indexing of qualitative studies was rather poor, and most of the references collected were actually quantitative studies. They were eliminated at this step. If the abstract was not sufficient, we read the entire article. Disagreements were resolved during meetings of the research group. The potentially relevant articles were then read in full, and a second selection made to keep only the article that met our inclusion criteria.

### Assessment of article quality

Evaluating the quality of the articles was an essential stage of this process, in order to discuss the studies' results and conclusions and judge the value and the integrity of the data used. To assess the quality of the qualitative articles, we used the Critical Appraisal Skills Program (CASP) [25]. The CASP comprises 10 questions: two screening questions about the aims of the research and appropriate use of a qualitative methodology, and eight questions covering research design, sampling strategy, data collection, researcher’s reflexivity, ethical issues, data analysis, the findings, and the value of the research. Two authors (JS and MO) performed this assessment independently and then discussed the results within the research group until we reached agreement. Given the lack of consensus about the role and function of study quality assessment as part of systematic reviews [15], we did not exclude any study from the analysis based on our evaluation. However, as noted by the original authors of the meta-ethnographic approach, studies of poorer quality tend to contribute less to the synthesis [15, 26].

### Data analysis

Our analysis followed the procedure described by Thomas and Harden [14], adapting them to the principles of the meta-ethnographic approach [15]. It began with an attentive reading and then repeated readings of the titles, abstracts, and texts of each article. One researcher (JS) extracted the formal characteristics of the studies, and three (JS, MO, and ARL) independently extracted and analyzed the first-order results (that is, the study results) and the second-order results (authors' interpretations and discussions of the results) of each study selected; these independent analyses were then compared and discussed at the research meetings. NVivo 10 qualitative analysis software was used to manage data and facilitate the development of themes.

Thematic analysis made it possible to develop themes inductively, from the study data. The work of *translation* involved comparing and assembling the themes obtained by the analysis of each article to retain the key themes that capture similar ideas in the different articles and then to develop overarching concepts about the research question. The high level of rigor of the results was obtained by triangulation of both the data sources and the analyses: three independent analyses and monthly research meetings to discuss the results [27].

## Results

### Presentation of the studies

**Fig 1:**
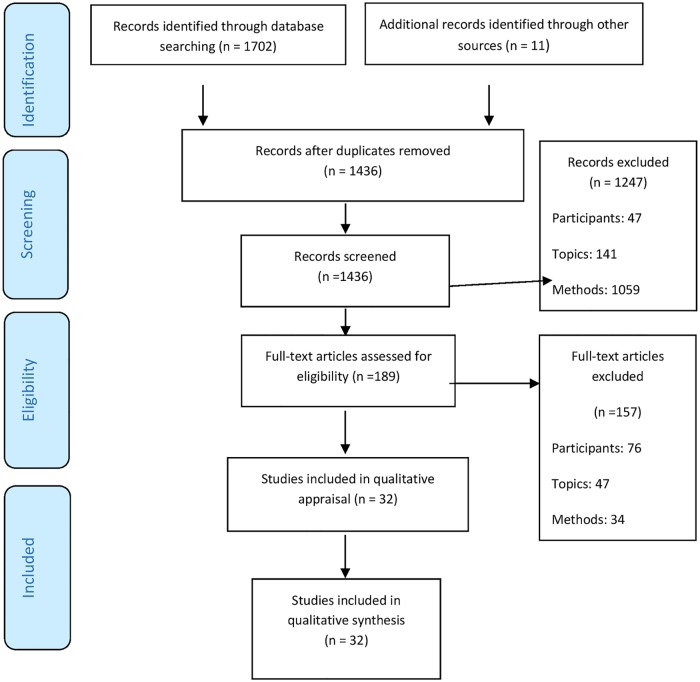
Flow of information through the different phases of the studies selection. *From*: Moher D, Liberati A, Tetzlaff J, Altman DG, The PRISMA Group (2009). *P*referred *R*eporting *I*tems for *S*ystematic Reviews and *M*eta-*A*nalyses: The PRISMA Statement. PLoS Med 6(6): e1000097. doi: 10.1371/journal.pmed1000097.

In all, we collected 1,713 references, 1,436 after the elimination of duplicates. We eliminated 1247 references when reading titles and abstracts. We read 189 complete articles and found only 32 that met our inclusion criteria, that is, 2.2% of the 1,436 articles initially obtained from our search (Fig 1). Specific problems concerned the studies that simultaneously included adolescent and young adult participants, as well as those mixing adolescents with various eating disorders. We applied our inclusion and exclusion criteria strictly.

We analyzed 32 studies, 25 of them published during the last decade. Data were collected most often by *semi-structured* or *in-depth* interviews, but we also found written documents, video recordings, and a focus group. Twelve studies included only adolescents with or recovered from AN, seven studies only parents, and six only health-care professionals (nurses, therapists, and treatment team). Five included both adolescents and their parents, and two both adolescents and the nurses caring for them. None of these 32 studies collected data simultaneously from samples of adolescents with AN, their parents, and their providers. Nonetheless the process of translating studies one into another, described above, enabled us to compare the perspectives of the adolescents, the parents, and the professionals about the treatment, and then to examine their differences and their similarities.

Sixteen studies took place in an inpatient setting only, nine in outpatient clinics, including five based on specific psychotherapeutic treatments, and one considered home treatment. These studies came from seven countries: 12 from Great Britain, ten from Australia, three from Canada, three from the Netherlands, two from Sweden, one from Ireland, and one from China. In all, 26 studies came from English-speaking countries. Table 2 describes the characteristics of each study.

**Table 2:** Main characteristics of the studies.

References	Year	Aim	Country	Population	Data collection	Method
Bakker et al. [28]	2011	To discover which aspects of nursing care are most effective in recovery of normal body weight in adolescents with anorexia nervosa (AN)	Netherlands	Nurses N = 8	Semi-structured interview (SSI)+ focus group	Thematic analysis
Ramjan [29]	2004	To explore the difficulties and obstacles hindering the formation of therapeutic relationships in this context between adolescents with AN and paediatric nurses.	Australia	Nurses N = 10	SSI	Thematic analysis + computer NUD*IST
Ramjan & Gill [30]	2012	To explore the experiences of adolescents in an inpatient behavioural program for the treatment of AN, as well as those of the nurses who cared for them.	Australia	Adolescents with AN age 11–18 N = 10 (Girls N = 9, boy N = 1); Nurses N = 10	SSI	Thematic analysis + Nvivo 2
Beukers et al. [31]	2015	To describe nursing interventions aimed at restoring normal eating behaviour in patients with AN.	Netherlands	Health professionals N = 8 Adolescents with AN age = 12–18 N = 9	Video recordings Direct Observation	Thematic analysis
Voriadaki et al. [32]	2015	To contribute to our understanding of the process of change that takes place in Multi-Family Therapy for adolescent anorexia nervosa.	United Kingdom	Adolescents girls with AN age 15–16 N = 5 and parents N = 10 (mother N = 6, father N = 4)	Focus group +daily journal	Interpretative phenomenological analysis
Zugai et al. [33]	2013	To establish how nurses ensure weight gain and a positive inpatient experience for the treatment of adolescents with AN.	Australia	Adolescents girls with AN age = 14–16 N = 8	SSI	Thematic analysis
Boughtwood & Halse [34]	2008	To examine how girls with AN negotiate and manage the dissonance between medical regimes and social discourse	Australia	Adolescent girls with AN age = 12–18 N = 25	SSI	Poststructural theory
Boughtwood & Halse [35]	2009	To identify how teenage girls diagnosed with AN construct their illness, treatment programs and relationships with their doctors and nurses	Australia	Adolescents girls with AN age = 12–18 N = 25	SSI + Field notes	Poststructural theory
Tierney [36]	2008	To explore the views of young people about being treated for AN	United Kingdom	Adolescents with AN age 11–18 N = 10 (Girls N = 9, boy N = 1)	SSI	thematic analysis + Atlas-ti
Offord et al. [37]	2006	To explore young adults’ views regarding the inpatient treatment they received for AN during adolescence.	United Kingdom	Young women N = 7 who had AN during adolescence	SSI	Interpretative Phenomenological Analysis (IPA)
Tierney [38]	2005	To find out parents’ views in relation to treatment received by their children with AN	United Kingdom	Parents of adolescent with AN N = 14	SSI	content analysis + Atlas-TI
King & Turner [39]	2000	To explore in depth the experiences of registered nurses caring for adolescent anorexic females on paediatric wards	Australia	Nurses N = 5	SSI	Colaizzi analysis
Van Ommen et al. [40]	2009	To develop a tentative theoretical model explaining the effectiveness of inpatient nursing care of adolescents with AN	Netherlands	Adolescents girls with AN, Age = 12–18, N = 13	SSI	Grounded theory
Colton & Pistrang [41]	2004	To provide a detailed description of how adolescents experience inpatient treatment for AN	United Kingdom	Adolescent girls with AN Age 12–17, N = 19	SSI	IPA
Freedman et al. [42]	2006	To explore the thematic content of the letters written by adolescents with AN to improve our understanding of how the adolescent population relates to the illness.	Canada	Adolescents with AN, mean age = 16.7, N = 27	27 Pro and 27 Con letters	Grounded theory
Koruth et al. [43]	2011	To explore young people’s experiences of the onset of AN.	United Kingdom	Adolescents with AN age 13–17 N = 8 (Girls N = 7, Boy N = 1)	SSI	Grounded theory
Nilsson & Hägglöf [44]	2006	To describe the patients’ perspective of the recovery process from AN.	Sweden	Previous adolescents with AN N = 68	SSI	Content analysis
Cottee-Lane et al. [45]	2004	To systematically describe parents’ experiences of having a child with AN.	United Kingdom	Parents of adolescents with AN N = 11	SSI	IPA
Sharkey-Orgnero [46]	1999	To explore parents’ views of their daughters’ recovery from AN	Canada	Mothers N = 10, fathers N = 8, recovered adolescents with AN N = 9, and sibling N = 1	SSI	Grounded theory
Easter & Tchanturia [47]	2011	To gain an understanding of therapists’ experiences of Cognitive Remediation Therapy with adolescents with AN through a qualitative analysis of the therapists’ end-of-treatment letters.	United Kingdom	Therapists N = 12	23 letters "end of treatment"	Grounded theory
McCormarck & McCann [48]	2015	To investigate the subjective experiences of parents in caring for an adolescent diagnosed with AN.	Ireland	Parents of adolescents with AN N = 10	SSI	Thematic analysis +Nvivo 9
Dallos & Denford [49]	2008	To explore the accounts of families regarding their emotional relationships, and to explore aspects of their accounts relating to attachment processes.	United Kingdom	4 families	SSI individual and joint interview	IPA + discourse analysis
Honey et al. [50]	2007	To address the question “What support do parents of teenage girls with AN want from clinicians?”	Australia	Parents of adolescent with AN N = 24	In-depth interviews	content analysis
Bezance & Holliday [51]	2014	To explore the experiences of mothers receiving home treatment as part of treatment for their daughters’ AN	United Kingdom	Mothers of adolescent with AN N = 9	SSI	IPA
Honey & Halse [52]	2007	To explore in detail the conscious and deliberate efforts that parents make to help well siblings adapt to AN in the family.	Australia	Parents of adolescents with AN N = 24	in-depth interviews	Grounded theory
Engman-Bredvisk et al. [53]	2015	To investigate Multi-Family therapy as part of AN treatment from a parental perspective	Sweden	Parents of adolescents with AN N = 12	SSI	Empirical psychological, phenomenological method
Honey et al. [54]	2006	To examine the ways siblings influence adolescent girls with AN	Australia	adolescents with AN Age = 14–20 N = 24 and parents N = 24	In-depth interview	Grounded theory
Jarman et al.[55]	1997	To examine the understandings and experiences of members of a community-based, multidisciplinary team of health-care professionals, whose work involves the treatment of young people with AN.	United Kingdom	Members of a child and adolescent team N = 7	SSI	IPA
Ma [56]	2008	To assess the effectiveness of family therapy as perceived by patients and families	China	Adolescents with AN, Mean age = 14 N = 18 + families	SSI	thematic analysis
Couturier et al. [57]	2013	To explore and describe therapists’ perceptions of the factors affecting their uptake of family-based treatment for adolescents with AN	Canada	Therapists N = 40	SSI	Conventional content analysis + Nvivo 8
Godfrey et al. [58]	2015	To develop insights into the processes taking place during family meal.	Australia	Adolescents with AN, Age = 12–18, N = 30 (girls N = 27, boys N = 3) and parents	Video recordings	Thematic analysis
Rich [59]	2006	To explore the ways in which adolescent girls manage the complexities of the presentation of an anorexic identity	United Kingdom	Adolescents girls with AN, Age = 11–17, N = 7	SSI + Field notes	ethnographic approach

### Quality assessment

The evaluation of the studies with CASP found good results on the whole. Nearly all (30) had scores of 7 or higher (out of 10). Ethical considerations were sometimes insufficient, as was author reflexivity, defined as the critical examination of the author's own role in every step of the research process. Editorial constraints might explain these absences (the maximum number of words allowed for many of these article was often more appropriate for quantitative studies). Table 3 summarizes the results of this analysis; the detailed presentation is included as supplemental material (S2 Table).

**Table 3:** CASP summary, by criterion.

Criteria	Example	Quality assessment of studies (summary)		
		Met criterion	Partially met	Did not meet
Aims	Explicitly stated aims/objectives of research	32	0	0
Method	Appropriate use of qualitative methods	32	0	0
Research Design	Justification of the specific research design	32	0	0
Sampling	Appropriate sampling strategy, description of recruitment, discussion around recruitment	27	3	2
Data collection	Appropriate description of data collection methods	30	1	1
Reflexivity	Critical examination of researchers' own role and potential bias in data collection and analysis	7	0	25
Ethical Issues	Evidence of approval by an appropriate body	13	0	19
Data Analysis	Adequate and in-depth description of analysis process, sufficiently rigorous data analysis	24	7	1
Findings	Clear statement of the findings, discussion of evidence, credibility, integrity	30	2	0
Value of Research	Contribution to existing knowledge, transferability	32	0	0

### Thematic analysis

Two central themes were inductively developed from the analysis: (1) the treatment targets, that is, on the one hand, the symptoms of AN and the aim to normalize behaviors, and, on the other hand, the subject with AN in her (or his) context, and (2) the relationship as a treatment tool, the only one reported by all of the parties involved, and especially the core concept of the therapeutic relationship. Table 4 presents quotations from participants and from the authors of primary studies for each theme.

**Table 4:** Quotations from participants and from authors of primary studies to illustrate each theme.

	Quotations from participants in primary studies		Interpretations of findings offered by authors
**Treatment targets**			
1. First Target: the symptoms of anorexia			
Observable target-symptoms	*Professionals*: Nurses unanimously perceived the adolescents as not having “what we would class as a normal eating habit or a normal view of food.” Most nurses saw their role as helping the adolescents return to normal eating patterns of “three square meals a day” [30]. *Adolescents*: "The staff didn’t really care what I did, as long as I put on weight” [36].	These interventions are described within the domains of resumption of a normal eating pattern and normal exercising, development of the patient’s social skills to facilitate social recovery, and supporting the parents in their full parent role [28]. The participants understood that weight gain was essential and that it required strict rules (…) an explicit and indiscriminate application of rules did not foster a positively perceived inpatient experience [32].	
Educating the adolescent about the disease	*Adolescents*: "Once I got out of hospital, she [mother] thought everything was back to normal and that I wasn’t purging and what not. And then I got depression. ‘Cause I hated the way I looked. Like, it [anorexia] might be the numbers [on the scale] but doesn’t the look also depend on the numbers as well?"[34].	The standard bio-medical indicators of physical well-being had limited persuasive power amongst girls in our study and they actively resisted the interpretation of their (anorexic) bodies by medical discourse [34].	
2. Second Target: the individual in context			
Adolescents' involvement in their own care	*Adolescents*: "I kind of felt like I was being heard and I actually had a part in this and wasn’t just a balloon being pumped up" [41]. *Adolescents*: " It was always just ‘and how has your week been?’… It wasn’t any good. …I would have liked it to go deeper" [36].	Several participants talked positively about inpatient treatment that did not focus solely on weight, shape and eating, but also addressed wider issues such as self-esteem, depressive thinking and systemic dynamics [37]. Participants spontaneously (and unanimously) expressed the view that the key to recovery was their own desire and readiness to get well [34].	
Consideration of the adolescent's social world	*Adolescents*: "Then you return home and see your friends again, you start to do things you used to do and also start to do fun things. After a while you were allowed to go out and notice that it is much more fun than having an eating disorder." [40]. *Professionals*: "To encourage them to learn new experiences, that life can be fun."[28].	We do emphasise that a comprehensive treatment program for adolescents with anorexia nervosa should also give sufficient (and balanced) attention to the emotional and psychosocial functioning [40].	
Consideration of the family	*Parents*: “Particularly at the beginning we were thinking why, why, what is it, and we changed absolutely everything we could possibly change but whether it was too late when we changed it all or whether it didn’t matter if we changed it or not I don’t know” [45].	Parents were puzzled about the causes of their child’s eating disorder but also articulated complex explanations of it [45].	
**A single therapeutic tool: the relationship**			
1. Variations of the relationship in treatment			
Role of Control in the relationship between the professionals and the adolescent	*Professionals*: “Someone else has to take that control away from the child” [55].	All units appeared to have set treatment programs for patients with anorexia, and these were often perceived as inflexible and punishing, especially when they were based on strict behavioural approaches [37].	
The relationship between adolescents and families	*Parents*: “I found it (family therapy) quite useful and I think the kids found it quite useful because they could say what they’d been bottling up for a long time.”[38].	(Adolescents) were more mixed in their opinions, welcoming family therapy as an arena for honesty and for explaining their feelings [36].	
Relationships between peers	*Adolescents*: “I think it was helpful, because it’s… you learn about, from other people, you learn how different people cope with things… so then you can take what they use and see if it works for you.”[37].	The study highlighted the value of positive peer relationships, a sense of community, and the opportunity to identify with others and learn positive ways of coping [37].	
2. The core concept of the therapeutic relationship			
The benefits of the therapeutic relationship	*Adolescents*: “so, when they give you that respect you feel like ‘ok, well, it’s not my fault I have this disorder like it’s just something I’ve got to get through’. And like, the way they’d show respect was probably by kind of giving you some kind of leniency with some things, not like with food or anything like that but like … they’d trust you…”[32]	For any nursing care plan to be effective, nurses and patients must be intimately involved in its development and evaluation [29]. A strong nursing relationship resulted in an improved inpatient experience [32]. The best outcomes are likely to be achieved through a cohesive approach where treatment providers and families work together [54].	
The conditions necessary for constructing a therapeutic relationship	*Parents*: “We were at the stage where we'd been [patient]’s mum and dad for 16 years, and we were used to working problems through with her, and then suddenly these barriers came down and we felt that things were happening to her and being discussed with her and that we were being blocked out of it.” [38].	Participants (nurses) believed in and based their nursing care on values that formed the core of their care of all patients. Equality of care, trust, privacy, being non-judgemental, maintaining confidentiality, assuring patients' rights and advocacy [39].	
Barriers to the construction of a therapeutic relationship	*Adolescents*: "it’s just like everything’s anorexic and everything you do’s anorexic…everyone always says you can’t trust an anorexic"[35]. *Professionals*:”I like to think that I can trust them and I find that you can't… you tend to be a bit cynical… And I guess, because I've seen so many of them sabotage their meals… I find I don't trust them as easy” [30].	Manipulation, mistrust and the struggle for control were the major obstacles to developing therapeutic relationships in these wards [29].	

### 1. Treatment targets

#### 1.1 First target: The symptoms of AN

This theme was found in all three groups and was predominant in the healthcare professionals' representation of treatment, for they relied on a biomedical discourse to define the target symptoms and their normalization. Professionals considered AN, which they viewed as a disease or disorder to be corrected, as the object of treatment. The point of treatment for them was to make the symptoms of AN disappear and allow the adolescent to return to normal. Professionals thus saw two aspects that should be distinguished: observable target-symptoms and patient education.

Observable target-symptoms: From the point of view of the professionals, treating AN was equivalent to normalizing the patient's weight, body, and behavior [28–30]. They encouraged the resumption of normal eating and the cessation of behavior intended to prevent weight gain [31]. The importance of physical care was stressed [30]. These aspects corresponded to the disease symptoms that could be observed from the exterior, symptoms that professionals could perceive and measure: the adolescent's weight, physical status, dietary behaviors, and hyperactivity [28,29].

Retrospectively, some adolescents recognized the importance of regaining weight and changing their behavior [32,33] but most of them criticized the method used and its effects. They denounced the use of the criterion of weight alone to judge health status and the course of care [30,34–36]. They also considered that the treatment focused too much on somatic aspects, while ignoring their psychological distress [36,37]. Parents shared this opinion and regretted that care focused too much on their child's physical health [38].

Educating the adolescent about the disease: Most professionals considered that patients must be educated about their disease to enable them to return to normal [28,30,31,39]. They sought to make the teens' anorexic cognitions disappear by informing them of the dangers of AN, especially its long-term risks [34,35]. The approach of the professionals was therefore situated in the adolescent's future [30,34,35].

Some adolescents valued the information they received, which improved their knowledge and their awareness of the disorder [40]. Other rejected the medical discourse and the objective criteria that determined health [35] or explained their confusion between the medical discourse focused on health and the social discourse focused on physical appearance [34].

#### 1.2 Second target: the individual in context

This theme was found in all three groups and was predominant in the adolescents' and parents' representations of treatment. Most adolescents insisted on the importance of individualized care, of being considered as unique people with their own individual, singular distress [36,37,40,41]. From the adolescents’ and parents’ points of view, above all, care should focus on the adolescents as individuals and complete people [36,37]. This holistic approach distinguished three aspects: the teens' involvement in their own care, consideration of their social world, and consideration of their families.

Adolescents' involvement in their own care: Most adolescents considered it essential to become the agents of their own care. They wanted to be involved in their care], to be responsible [32,37,40,42]. For them, their care took place in the present moment and they insisted on the experience of care on a daily basis [40,42]. They considered their own desire for recovery as an essential element of care and of the recovery process [33,36,37,41,43,44].

The parents' representations were similar: they considered that their child's lack of motivation for change frustrated all their efforts [28,45,46].

Some professionals underlined the importance of mirroring the adolescents' attitudes and perceptions back at them in a motivational approach [28,31], or of developing their metacognitive capacities in therapy, that is, of helping them to understand their own thoughts better [47]. The objective was to enable the adolescents to care for themselves, autonomously [28].

Consideration of the adolescents' social world: Most adolescents also considered that the treatment targets must take their social life into account. Maintenance of associations with the outside world during hospitalization was a necessary part of their treatment [30,36,37]. During the hospitalization, some nurses sought to ensure that links with the outside world continued [28], while other nurses and therapists sought to develop the teens' social skills [28,47].

Consideration of the family: Adolescents and parents believed that management of family distress should be an integral part of the treatment [48] and that the professionals should care for the entire family, including the siblings [38,49,50].

The parents felt confused, frustrated, and frightened [45,48,51], guilty and powerless to help their child [38,45,46,48]. They were especially overwhelmed by the question of “why”: the origins and causes of the child's AN. For them, treatment required an exploration of the past [38,45,52]. It was difficult for them to find support from their family or friends or from healthcare professionals [45,52]. They expected the professionals treating the child to provide all of them with systematic support: a look back at the reasons for the disease, practical advice specifically individualized to their family, emotional support, and aid in meeting parents in the same situation, either in support groups or in a multi-family therapy setting [45,48,52,53,54].

### 2. A single therapeutic tool: the relationship

#### 2.1 Variations of the relationship in treatment

The relationship was central in the discourse of all the protagonists and varied during treatment according to whether it involved a relationship with the professionals, or that with the family or that between peers.

Role of control in the relationship between professionals and adolescents: For most professionals, the therapeutic relationship with adolescents included an aspect of control. They considered it necessary to assume control of the adolescents' actions to enable normalization and the disappearance of symptoms. They believed that they must decide in the patients' place [28,55] and maintain a framework, structured by the department's rules and protocols [29,30]. Some professionals tried to balance their controlling approach with kindness [31] but, most of the time, this takeover induced a power struggle, described both by the professionals [28,29,39,55] and the teens [34,35,37,41].

Adolescents were ambivalent about this relational control. On the one hand, they recognized the therapeutic importance of the continuous surveillance [33,41,42]. On the other hand, they experienced it as coercion and punishment [29,30, 34,37,40,41].

The relationship between adolescents and families: Intrafamily relationships were dealt with as part of family therapy. This process enabled the parents to work on family interactions and family history [38,56]. This therapy also enabled the family to play an active role in care [56,57,58]. Parents described it as a space for expression, sharing emotions, identifying family conflicts, and reconstructing a bond of trust [38,51,56]. It was also considered by both the teens and their parents as the best way to involve the siblings in this care [54, 58].

Relationships between peers: Adolescents with AN also reported the importance of their interactions with each other and noted that their relationships with other teens with AN have important effects—both positive and negative [38,37,40,41,59]. On the one hand, friendships between adolescents with AN enabled support and mutual understanding [31,37,59]. the other hand, comparison of behaviors could lead to reinforcing symptoms [36,37,41]. The adolescents with AN also reported group phenomena, either caring for themselves and recovering from AN together [40] or resisting treatment together [34].

#### 2.2 The core concept of therapeutic relationship

Adolescents, parents, and professionals all considered the therapeutic relationship as the core concept for ensuring the effectiveness of treatment. Indeed, they had the same vision of the benefits of a good therapeutic relationship and about the conditions for constructing a therapeutic relationship. Finally, in practice, they experienced the same barriers to establishing a good therapeutic relationship: mutual distrust and lack of communication.

The benefits of the therapeutic relationship: Adolescents, parents, and professionals all recognized the importance of constructing a good therapeutic relationship.

Most adolescents considered this relationship to be essential to treatment [33,35,41,43], to allow them to experience care as a collaboration between themselves and the professional [33,37,41]. A relationship of trust was experienced as therapeutic [33,36,40]. Some adolescents pointed out their desire for a special relationship with a professional able to inspire motivation and adherence to the treatment plan [33,40].

For health-care providers, establishing a therapeutic relationship with the adolescents was the major challenge in treating them [28–30,39]. The professionals considered relationships with the parents important as well: effective treatment of the child is impossible unless parents are supportive and invested in the treatment [28,46,57]. According to them, including parents in the "recovery team" can enhance the teen's therapeutic relationship and improve care [46].

Parents reported that three actions appear necessary to guarantee a trusting relationship between the professionals and themselves: the professionals must support them [40,48–50], involve them [45,46,53], and inform them [33,46].

The conditions necessary for constructing a therapeutic relationship: All parties considered that the health professional's empathy and humanist values are necessary preconditions to building a good therapeutic relationship.

Adolescents with AN expected human qualities and ethical care from the professionals caring for them [33,34,36,40,41,43]. They should be empathetic, understanding, accessible, and available, thoughtful, humble, and reliable [33,36,40,41,43]; they must not be judgmental [34,36].

The professionals based their practices on these same humanist values: understanding, empathy, respect, equality in care, and a non-judgmental approach [28,31,39]. They felt responsible and sought to demonstrate reflexivity in their practice [29,39,55].

Parents expected the same values: sincerity, respect, and empathy [38,52,56]. Some parents stressed the importance of recognizing the uniqueness of their situation [52].

Barriers to the construction of a therapeutic relationship: Nonetheless, according to the studies included in our synthesis, adolescents, parents, and professionals seemed unaware that they shared this same conception of the therapeutic relationship and of these relational values. *Mutual distrust* [29] developed in place of the desired mutual trust, especially between the adolescents and their healthcare providers [29,39]. This mutual distrust impeded the establishment of the therapeutic relationship.

The adolescents were suspicious [29,30] and considered that the professionals lacked empathy and understanding, that they had prejudices and a negative opinion of patients with AN [28,29].

The professionals explained that they became distrustful and suspicious in reaction to the adolescents' opposition and resistance to treatment [29,39]. They became unable to trust patients whom they found to be manipulators and liars [29,39]. They made subjective judgments, showed favoritism, and stigmatized the adolescents as *“anorexic”*, thereby indicating a preference for patients with disorders other AN [29,30].

As for the parents, they also felt "de-skilled" as parents by the professionals [38,45] and complained about a lack of confidence in them [52].

## Discussion

This metasynthesis gave us access to how adolescents with AN, parents, and professionals experience the treatment of AN in adolescence. In our first central theme, we found disagreement in the representations of the targets of AN treatment, especially between the healthcare providers and the adolescents. For the professionals, the disease of AN is the target, with its various symptoms; the goal is thus for AN, the disease, to disappear, through normalization of the teen's weight and behavior. This approach is seen in the literature, where some studies about recovery and therapeutic response in AN refer only to physical and behavioral criteria [5,60]. Moreover, some authors have argued that targeting the disease distinguishes the individual from the symptoms and should thus reduce the guilt of both adolescents and parents [57]. For others, returning to a normal Body Mass Index (BMI) is also considered a necessary prerequisite to the ability to modify anorexic cognitions [61].

For the teens included in the studies, however, the target of the treatment appears be the subject—that is, themselves; it must consider their psychological and social functioning and their family environment. This belief is consistent with the position of the many authors who have underlined the need to integrate these psychological and social dimensions to enable complete recovery of patients with AN [62]. It is also consistent with the *recovery model*, an approach based on a holistic view of mental illness that focuses on the subject and aims to help people with mental illness achieve personally meaningful goals [63]. Unfortunately, up until now, the illness management and recovery process associated with this approach has been used mostly with people with schizophrenia or bipolar disorder and has not yet been widely implemented in the area of eating disorders.

The differences between adolescents and professionals in their definitions of treatment targets, shown by our results, mirror the discordances in the literature about the definition of recovery in adolescents with AN [64]. Several studies have demonstrated the risks of defining recovery only by physical and behavioral criteria and not taking the individual’s psychological functioning into account. The persistence of anorexic cognitions and food restriction after normalizing weight [65] and the high levels of depression, anxiety, and obsessional behavior among adolescents who have “recovered” from AN [66] may produce instead a *pseudorecovery*, that is, a physical recovery but with the psychological components of AN unchanged [67] and therefore a high risk of relapse [68].

Moreover, by aiming at these different targets, each group focuses on a different time period and a different time scale. More precisely, (i) the parents target family history and focus on the past; (ii) the adolescents anchor treatment in the day-to-day of the present; and (iii) the professionals anticipate risks in the future.

Although the treatment targets differ, the protagonists all agree that the therapeutic relationship is an essential tool of treatment. It is thus the second central theme of our results. All of them underline the relational dimension of care, especially based on their shared ideas of the therapeutic relationship. Our results show, however, that despite this shared vision adolescents, parents, and professionals all encounter barriers to its construction. The disagreement about the targets together with the mutual mistrust described in our results is highly likely to impede the construction of a good therapeutic relationship. That is, the adolescents and their healthcare providers distrust each other and each focus on different treatment targets. This distrust and these different goals create the risk that no dialogue about the treatment will take place, that no shared communication space can emerge around the treatment, and that neither will know the viewpoint of the others about this topic.

### Clinical and research implications

These concepts of treatment targets and therapeutic relationships are related to the more general issues of the therapeutic alliance. Bordin [69] has defined the therapeutic alliance as a mutual understanding and agreement about the goals for change and the tasks necessary to advance toward these goals while awaiting the creation of bonds to support the work of all involved. In a recent qualitative study, adolescents with AN identify therapeutic alliance as one of the most helpful aspect of the treatment [70]. The literature also includes several studies that stress the importance of establishing this alliance in treating AN in adolescents, in terms of outcomes in hospitals and institutions [71] and in individual and family psychotherapy [72–74] and of dropout rate [75,76]. Other factors have also been described as predictors of better outcome, in particular, early improvement in symptoms and an ego-dystonic experience of eating disorders [77,78]. Our results shed light on the process of the therapeutic alliance and encourage us to consider it as an important lever of treatment for AN in adolescents. They suggest that dialogue between adolescents, parents, and professionals about treatment—its targets and the barriers to a therapeutic relationship—is an essential element in the process of forging a therapeutic alliance for this treatment. Each stakeholder must know the others’ views of its aims to reach to an agreement and a mutual understanding about their “joint” care project.

Nonetheless assessment of dialogue is missing from the instruments that measure therapeutic alliance in adolescents. These scales have most often been developed from those used to measure this alliance in adults. They are not specific to the adolescent population and could be used only after modification to include parents and to adapt the description of items so that they are understandable by adolescents. Accordingly, neither the Adolescent Working Alliance Inventory (AWAI) [79] nor the Helping Alliance Questionnaire for Children and Parents (HAQ-CP) [80], both used in studies of AN in adolescents [71,73], includes an item covering the dialogue between adolescents, parents and healthcare professionals, nor do they include any item exploring whether each knows and understands the others' positions about the treatment. The concept of therapeutic alliance cannot be reduced solely to mutual agreements about care and to the interpersonal relationships between adolescents and healthcare providers; it must also take into consideration the existence of a space for dialogue between the protagonists, where divergences and disagreements can be articulated to enable each party to know the other's position. The existing scales measure a result of alliance without approaching its process. This may be because little is known about the specificities of this process of therapeutic alliance in adolescent psychiatry generally and in the treatment of AN in particular. Neither its establishment nor the factors that can facilitate or impede it have been described or studied. Our results here provide some initial elements about the need for a space of authentic dialogue about care to achieve an alliance and build such a shared space. We think that further research, first qualitative and then psychometric, is needed to explore the processes involved in establishing a therapeutic alliance in this situation and to create specific measurement tools for this population and this disease.

### Strengths and limitations

This review integrates the views about treatment of AN in adolescence from 322 adolescents with AN, 164 of their parents, and 109 healthcare professionals, including 41 nurses. The method we applied is rigorous, tested in medical research [14,15], and meets the criteria of the ENTREQ guidelines [18]. We analyzed 32 articles, all published in peer-reviewed journals and mostly of good quality. Even though no qualitative study has collected data simultaneously about adolescents, parents, and professionals, this synthesis crossed the views of all three about treatment of AN in adolescents and has provided perspectives much broader than any of the initial studies.

However, certain methodological and clinical aspects of this metasynthesis limit the generalization of its conclusions. A qualitative metasynthesis collects only partial data from the participants and the interpretations of the researchers, which are the data given in the initial articles. We therefore lack precise data about the type of treatment these young people received. Nonetheless, the objective of this metasynthesis was to explore the experience of care and not to compare its content.

Moreover, the qualitative data collected in this study come from diverse and heterogeneous therapeutic models. More than half the studies explored the experience of inpatient treatment. Hospitalized adolescents with AN are more likely to have cognitive impairment and to resist treatment [7], both factors that may influence the nature of treatment and the interactions between patients, parents, and professionals. The role of parents in inpatient care depends on the protocols applied in these units. The most delicate period of care is that of inpatient hospitalization, which involves significant concerns about treatment adherence and the risks of dropping out or relapse. The stakes of the therapeutic alliance that our results underline are therefore especially salient. Nonetheless, our methodological approach makes it possible to decontextualize the initial qualitative data [13].

In view of the heterogeneity of the possible treatments, we have chosen to restrict the clinical population to those with of AN alone, including its restrictive and its binge and purge forms. In most of the articles studies, the populations met DSM-IV criteria. This choice enabled us to analyze a corpus homogenous in its overall topic. Nonetheless, we were not able to include studies with a sample of patients with transdiagnostic eating disorders or those exploring within-patient migration between eating disorder diagnostic categories.

Several articles included must probably come from the same original studies [34,35,50,52,54]. Although these papers explore different aspects of the experience of treatment in anorexia nervosa, there is a risk that they exert a greater weight than others in our results. In addition, we note that although the review includes articles from diverse cultural areas but with articles from English-speaking countries being overrepresented. It is nonetheless likely that the literature accessible by these international databases would overrepresent English-speaking countries even if we had selected articles in all languages.

Finally, boys with AN are underrepresented in this review since only four studies included boys [30,36,43,58]. Thus, our results may not be relevant for the male population. Further, a qualitative study exploring the boys’ views about treatment is needed to determine the similarities and discrepancies with the girls.

## Conclusion

This metasynthesis has enabled us to envisage clinical implications about the role of the dialogue between adolescents with AN, their parents, and their healthcare providers about treatment targets and about the barriers to therapeutic relationships. Within this dialogue, each stakeholder would know the objectives of the others and thus be able to build the shared that are an essential component of any therapeutic alliance [68]. This dialogue accordingly appears to be an essential element of the process of forging a therapeutic alliance for the treatment of adolescents with AN.

Our results also led to some research perspectives about the processes involved in establishing a therapeutic alliance in this situation and the need for specific measurement tools for this population and this disease.

## Supporting Information

S1 File ENTREQ Guidelines. Y = Criterion met; P = Criterion partially met; N = Criterion not met.
(DOCX)

S1 Table Complete search strategy for each database. Performed between March 01, 2014 and April, 15 2014. Updated September 2015.
(DOCX)

S2 Table Evaluation of study quality of the 32 studies included, according to the Critical Appraisal Skill Program (CASP). (DOCX)
