# Supplementary material for: Metasynthesis of the Views about Treatment of Anorexia Nervosa in Adolescents: Perspectives of Adolescents, Parents, and Professionals
Source: PLoS One. 2017 Jan 5;12(1):e0169493. doi: 10.1371/journal.pone.0169493 (PMC5215824; doi:10.1371/journal.pone.0169493)
Supplement: S1 File — Y = Criterion met; P = Criterion partially met; N = Criterion not met. (DOCX) [file pone.0169493.s001.docx]

| **S1 File. ENTREQ Guidelines**  Tong et al.: Enhancing transparency in reporting the synthesis of qualitative research: ENTREQ. BMC Medical Research Methodology 2012 12:181. | | |
| --- | --- | --- |
| No | Item | Rationale |
| 1 | Aim | To describe, compare, and contrast the perspectives of adolescents with anorexia nervosa, parents and professional about treatment of anorexia nervosa |
| 2 | Synthesis methodology | relies on the model of meta-ethnography and follows the procedures of the thematic synthesis |
| 3 | Approach to screening | Comprehensive search strategies |
| 4 | Inclusion criteria | - Papers in English - Qualitative methodology - Published between 1990 and 2015 - Studies that examined the views about treatment of the people involved in anorexia nervosa during adolescence — patients with AN (younger than 18 years during their disease), parents, and healthcare professionals. |
| 5 | Data sources | Electronic database (Medline, PsycINFO, CINAHL, EMBASE, SSCI) |
| 6 | Electronic Search Strategy | See supplemental material S1 |
| 7 | Study Screening Methods | After collecting the references and eliminating duplicates, two authors subsequently read the titles and abstracts to assess their relevance to our subject and their methodology according to our inclusion and exclusion criteria. If the abstract was not sufficient, we read the entire article. Disagreements were resolved during meetings of the research group. The potentially relevant articles were then read in full, and a second selection made to keep only the articles that met our inclusion criteria |
| 8 | Study Characteristics | See Table 2 in the main manuscript |
| 9 | Study selection results | See Figure 1 ( Flow of information through the different phases of the studies selection) |
| 10 | Rationale for appraisal | Critical Appraisal Skills Program (CASP) |
| 11 | Appraisal items | See Table 3 |
| 12 | Appraisal process | Two authors performed this assessment independently and then discussed the results within the research group until agreement was reached. |
| 13 | Appraisal results | See Table 3 and Supplemental material S2 |
| 14 | Data extraction | Three authors attentive read and re-read the titles, abstracts, and texts of each article. One researcher extracted the formal characteristics of the studies, and three independently extracted and analysed the first-order results (that is, the perspective of the participants in the original studies, most often reported in the results section) and the second-order results (authors' interpretations and discussions of the results, most often reported in the discussion/conclusion section) of each study selected |
| 15 | Software | Nvivo 10 Software |
| 16 | Number of reviewers | 3 |
| 17 | Coding | Coding by “unit of meaning” to search for concepts and ideas |
| 18 | Study comparaison | **Translation** = comparing and assembling the themes obtained by the analysis of each article to retain the key themes that capture similar ideas in the different articles and to develop overarching concepts about the research question |
| 19 | Derivation of themes | Inductive |
| 20 | Quotations | See Table 4 (Quotations from participants and from authors of primary studies to illustrate each theme) |
| 21 | Synthesis output | See Discussion section |
