# Supplementary material for: Metasynthesis of the Views about Treatment of Anorexia Nervosa in Adolescents: Perspectives of Adolescents, Parents, and Professionals
Source: PLoS One. 2017 Jan 5;12(1):e0169493. doi: 10.1371/journal.pone.0169493 (PMC5215824; doi:10.1371/journal.pone.0169493)
Supplement: S1 Table — Performed between March 01, 2014 and April, 15 2014. Updated September 2015. (DOCX) [file pone.0169493.s002.docx]

**S1 Table**. **Complete search strategy for each database.** Performed between March 01, 2014 and April, 15 2014. Updated September 2015.

| **Medline (Pubmed) (1948-)**  (“qualitative research” [Mesh] OR “Nursing Methodology Research” [Mesh] OR “Focus Groups” [Mesh] OR “observation” [Mesh] OR “qualitative research” OR “qualitative study” OR “qualitative method”) AND (perception OR attitude OR feeling OR knowledge OR belief OR view OR perspective OR opinion OR experience OR image OR "self concept" OR barrier* OR psycholog* OR management OR organization* OR "Attitude of Health Personnel"[Mesh] OR "Attitude to Health"[Mesh] OR "Knowledge"[Mesh] OR "Psychology"[Mesh] OR "Self Concept"[Mesh] OR "Health Services Administration"[Mesh]) AND (“Anorexia” [Mesh] OR “anorexia nervosa” [Mesh] OR “underweight” [Mesh] OR “thinness” [Mesh] OR “body dysmorphic disorder” OR “food refusal” OR “restrictive eating disorder”)  AND (“Adolescent” [Mesh] OR “Young Adult” [Mesh] OR “Adolescent Psychology” [Mesh] OR “Adolescent Psychiatry” [Mesh] OR “Adolescent Behavior” [Mesh] OR “Adolescent Development” [Mesh] OR “teenagers” OR “teens” OR “adolescence” OR “adolescent” OR “adolescents” OR “young”) |
| --- |
| **PsycINFO (EBSCO Publishing) (1800-)**  (DE "Qualitative Research" OR DE "Interviews" OR DE "Intake Interview" OR DE "Interview Schedules" OR DE "Psycho diagnostic Interview" OR DE "Grounded Theory" OR DE "Observation Methods" OR DE "Ethnography" OR DE "Discourse Analysis" OR DE "Content Analysis" OR DE "Phenomenology" OR DE "Philosophies" OR DE "Constructivism" OR DE "Hermeneutics" OR DE "Narratives" OR DE "Biography" OR DE "Life Review" OR DE "Storytelling" OR "qualitative research" OR "qualitative study" OR "qualitative method” OR "qualitative research" OR "qualitative study" OR "qualitative method") AND (DE "Attitudes" OR DE "Knowledge (General)" OR DE "Psychology" OR DE "Management" OR psycholog* OR feeling OR attitude OR knowledge OR view OR perspective OR opinion OR experience OR image OR "self concept" OR barriers OR management OR organization*) AND (DE “Anorexia nervosa” OR DE “food refusal” OR DE “underweight” OR DE “[Body Dysmorphic Disorder](javascript:XslPostBack('ctl00$ctl00$MainContentArea$MainContentArea$xslResults','ThesaurusLink','LinkTarget%7CauthorityList%24LinkTerm%7CDE%2B%2522Body%2BDysmorphic%2BDisorder%2522');)” OR “anorexia nervosa” OR “Anorexia” OR “food refusal” OR “underweight” OR “thinness” OR “restrictive eating disorder”) AND (DE “Adolescent Psychiatry” OR DE “Adolescent Psychology” OR DE “Adolescent Psychopathology” OR DE “Adolescent Psychotherapy+” OR DE “Adolescent Attitudes” OR DE “Adolescent Development” OR “teenagers” OR “teens” OR “adolescence” OR “adolescent” OR “adolescents” OR “young”)  Limits: Humans, Journal Articles |
| **CINAHL Plus - Cumulative Index to Nursing and Allied Health Literature (EBSCO Publishing) (1981-)**  ((MH "Qualitative Studies+") OR (MH "Focus Groups") OR (MH "Interviews+") OR (MH "Narratives") OR (MH "Observational Methods+") OR (MH "Discourse Analysis") OR (MH "Thematic Analysis") OR (MH "Semantic Analysis") OR (MH "Field Studies") OR (MH "Audio recording") OR (MH "Constant Comparative Method") OR (MH "Content Analysis") OR (MH "Field Notes") OR "qualitative research" OR "qualitative study" OR " qualitative method") AND ((MH "Attitude+") (MH "Knowledge+") (MH "Self Concept+") (MH "Psychology+") (MH "Management+") feeling OR attitude OR knowledge OR view OR perspective OR opinion OR experience OR image OR "self concept" OR barriers OR management OR organization* OR psycholog*) AND ((MH "Anorexia Nervosa") OR (MH “Body Dysmorphic Disorder”) OR (MH “Anorexia”) OR (MH “thinness”) OR (“Anorexia*”) OR (“Anorexia nervosa”) OR (“food refusal”) OR (“underweight”) OR (“thinness”) OR (“Body Dysmorphic Disorder”) OR (“restrictive eating disorder”)) AND ((MH “Adolescence+”) OR (MH “Adolescent Care”) OR (MH “Adolescent Health”) OR (MH “Adolescent Psychiatry”) OR (MH “Adolescent Psychology”) OR (MH “Adolescent Development”) OR (MH “Adolescent Behavior”) OR (“teenagers”) OR (“teens”) OR (“adolescence”) OR (“adolescent”) OR (“adolescents”) OR (“young”))  Limits: Humans, Journal Articles |
| **SSCI – Social Sciences Citation Index (Web of Science) (1956-)**  (“case study” OR “constant comparative” OR “content analysis” OR “descriptive study” OR “discourse analysis” OR (ethnography OR ethnographic) OR (“Focus group” OR "focus groups") OR “grounded theory” OR interview* OR narrative* OR observation* OR “qualitative method*” OR “qualitative research” OR “qualitative study” OR “thematic analysis” OR “semi-structured” OR “in depth”) AND (“anorexia nervosa” OR “anorexia” OR “food refusal OR “thinness” OR “underweight” OR “restrictive eating disorder” OR “body dysmorphic disorder”)  AND (perception OR attitude OR feeling OR knowledge OR belief OR view OR perspective OR opinion OR experience OR image OR "self concept" OR barrier* OR psycholog* OR management OR organization*) AND (“teenagers” OR “teens” OR “adolescence” OR “adolescent” OR “adolescents” OR “young”)  Limits: Humans, Articles |
| **Embase (Ovid) (1974-)**  ('qualitative research'/exp OR 'narrative'/exp OR 'observational study'/exp OR 'thematic analysis'/exp OR 'content analysis'/exp OR 'constant comparative method'/exp) AND (‘anorexia nervosa'/exp OR ‘body dysmorphic disorder’/exp OR ‘anorexia'/exp OR ‘underweight'/exp OR anorexia nervosa OR anorexia OR food refusal OR underweight OR thinness OR restrictive eating disorder OR body dysmorphic disorder)  AND ('attitude'/exp OR 'knowledge'/exp OR 'psychology'/exp OR 'self concept'/exp OR 'management'/exp OR experience OR organization*)  AND (‘adolescent'/exp OR ‘child behavior'/exp OR ‘adolescent development'/exp OR ‘adolescent disease'/exp OR ‘adolescent health'/exp OR ‘child psychiatry'/exp OR ‘child psychology'/exp OR teenagers OR teens OR adolescence OR young)  Limits:  Advanced Search (options accepted):  Map to preferred terminology (with spell check)  Also search as free text  Include sub-terms/derivatives (explosion search)  Records from: Embase  Quick limits: Humans  Type: Article |
