# Supplementary material for: Metasynthesis of the Views about Treatment of Anorexia Nervosa in Adolescents: Perspectives of Adolescents, Parents, and Professionals
Source: PLoS One. 2017 Jan 5;12(1):e0169493. doi: 10.1371/journal.pone.0169493 (PMC5215824; doi:10.1371/journal.pone.0169493)
Supplement: S2 Table — (DOCX) [file pone.0169493.s003.docx]

**S2 Table**. **Evaluation of study quality of the 32 studies included, according to the Critical Appraisal Skill Program (CASP).** Y =Criterion met; P=Criterion partially met; N= Criterion not met

| References | Aims | Methods | Research design | Sampling | Data collection | Reflexivity | Ethical issues | Data analysis | Findings | Value of reseach |
| --- | --- | --- | --- | --- | --- | --- | --- | --- | --- | --- |
| Bakker et al., 2011 [28] | Y | Y | Y | N | Y | N | Y | Y | Y | Y |
| Ramjan, 2004 [29] | Y | Y | Y | Y | Y | Y | Y | Y | Y | Y |
| Ramjan & Gill, 2012 [30] | Y | Y | Y | Y | Y | N | Y | Y | Y | Y |
| Beukers et al., 2015 [31] | Y | Y | Y | Y | Y | N | Y | Y | Y | Y |
| Voriadaki et al.,2015 [32] | Y | Y | Y | P | Y | N | N | Y | Y | Y |
| Zugai et al., 2013 [33] | Y | Y | Y | Y | Y | N | Y | Y | Y | Y |
| Boughtwood & Halse, 2008 [34] | Y | Y | Y | Y | Y | Y | N | P | Y | Y |
| Boughtwood & Halse, 2009 [35] | Y | Y | Y | Y | Y | Y | N | P | Y | Y |
| Tierney, 2008 [36] | Y | Y | Y | Y | Y | N | N | Y | Y | Y |
| Offord et al., 2006 [37] | Y | Y | Y | Y | Y | N | Y | Y | Y | Y |
| Tierney, 2005 [38] | Y | Y | Y | Y | Y | Y | N | Y | Y | Y |
| King & Turner, 2000 [39] | Y | Y | Y | Y | Y | N | Y | Y | Y | Y |
| Van Ommen et al., 2009 [40] | Y | Y | Y | Y | P | N | Y | Y | Y | Y |
| Colton & Pistrang 2004 [41] | Y | Y | Y | Y | Y | N | N | Y | Y | Y |
| Freedman et al., 2006 [42] | Y | Y | Y | Y | Y | N | N | Y | P | Y |
| Koruth et al., 2012 [43] | Y | Y | Y | P | Y | Y | N | Y | Y | Y |
| Nilsson & Hägglöf, 2006 [44] | Y | Y | Y | Y | Y | N | N | P | Y | Y |
| Cottee-Lane et al., 2004 [45] | Y | Y | Y | Y | Y | N | N | Y | Y | Y |
| Sharkey-orgnero, 1999 [46] | Y | Y | Y | Y | Y | N | N | Y | Y | Y |
| Easter & Tchanturia, 2011 [47] | Y | Y | Y | Y | Y | N | N | N | Y | Y |
| McCormack & McCann, 2015[48] | Y | Y | Y | Y | Y | N | Y | Y | Y | Y |
| Dallos & Denford, 2008 [49] | Y | Y | P | Y | N | N | N | P | P | Y |
| Honey et al., 2007 [50] | Y | Y | Y | Y | Y | N | N | Y | Y | Y |
| Bezance & Holliday, 2014 [51] | Y | Y | Y | N | Y | Y | Y | P | Y | Y |
| Honey & Halse, 2007 [52] | Y | Y | Y | Y | Y | N | N | Y | Y | Y |
| Engman-Bredvik et al., 2015 [53] | Y | Y | Y | Y | Y | N | Y | Y | Y | Y |
| Honey et al., 2006 [54] | Y | Y | Y | Y | Y | N | N | Y | Y | Y |
| Jarman et al., 1997 [55] | Y | Y | Y | Y | Y | N | N | Y | Y | Y |
| Ma, 2008 [56] | Y | Y | Y | N | Y | N | N | P | Y | Y |
| Couturier et al., 2013 [57] | Y | Y | Y | Y | Y | N | Y | P | Y | Y |
| Godfrey et al., 2015 [58] | Y | Y | Y | Y | Y | N | Y | Y | Y | Y |
| Rich, 2006 [59] | Y | Y | Y | Y | Y | Y | N | Y | Y | Y |
